# Effect of a maternal counselling intervention delivered by community health workers on child nutrition: secondary analysis of a cluster randomised controlled trial in India

**DOI:** 10.1186/s12889-021-11998-w

**Published:** 2021-11-05

**Authors:** Israa Alzain Ali, Arun Shet, Maya Mascarenhas, Maria Rosaria Galanti

**Affiliations:** 1 Department of Global Public Health, Karolinska Institute, Solnavägen 1E (Torsplan), 11365 Stockholm, Sweden; 2 Sickle Cell Branch, National Heart Lung and Blood Institute, National Institutes of Health, Bethesda, Maryland USA; 3 MYRADA, Bangalore, India; 4 Department of Global Public Health, Karolinska Institute, Centre for Epidemiology and Community Medicine (CES), Stockholm County’s Health Care District (SLSO), Stockholm, Sweden

**Keywords:** Counselling, Child nutrition, Dietary intake, Maternal knowledge, Cluster RCT

## Abstract

**Background:**

India suffers from a double burden of malnutrition and anaemia. The Karnataka anaemia project indicated that a counselling intervention delivered by community health workers improved anaemia cure rates.

**Objective:**

To evaluate the effect of maternal counselling on nutritional aspects of anaemia prevention.

**Methods:**

Secondary analysis of a cluster randomised controlled trial (55 simultaneously randomised villages using random number generator in Chamrajnagar district, Northern India). In the intervention group mothers of anaemic children received five monthly counselling sessions plus usual care (iron and folic acid supplements), while mothers of anaemic children in the control group received usual care alone. Daily intake of nutrients related to anaemia prevention, i.e. iron (mg) and vitamin C (mg), was estimated using the 24-h dietary recall method at baseline and 6 months follow-up. Linear and logistic mixed regression models were used to assess between-groups difference in changes in nutrients intake from baseline to end of follow-up. Data collectors and analysts were blinded to the group assignment.

**Results:**

Participants were 534 (intervention *n* = 303; usual treatment *n* = 231) anaemic children, aged 1 to 5 years and their caregivers, of whom 521(intervention *n* = 299 from 28 villages; usual treatment *n* = 222 from 27 villages) were retained at 6 months follow-up and included in the analysis. This study provides inconclusive evidence of improvement in the intake of nutrients that prevent anaemia from baseline to follow-up among the intervention compared to the control group; increase in iron intake was 0.24 mg/day (95% CI -0.67; 1.15) and increase in vitamin C intake was 4.61 mg/day (95% CI -0.69, 9.91). Although encouraging, it is notable that the overall intake of nutrients that prevent anaemia remained well below the national recommended daily allowance.

**Conclusion:**

This study provides inconclusive evidence of the effect of parental counselling on nutritional aspects of anaemia prevention. The results highlight the need to devise multi-component anaemia-prevention interventions that include facilitators of the availability of nutritious food and should be evaluated in studies that are adequately powered to detect nutritional changes.

**Trial registration:**

International Standard Randomized Controlled Trial Number ISRCTN68413407, prospectively registered on 17/12/2013.

**Supplementary Information:**

The online version contains supplementary material available at 10.1186/s12889-021-11998-w.

## Background

The global statistics for undernutrition remain alarming with 150 million children stunted and 50 million children wasted in 2018 [1]. In fact, undernutrition still accounts for 45% of the under-five mortality worldwide [1]. The global nutrition report highlights the need to intensify efforts to meet the second sustainable development goal (SDG) of ending all forms of malnutrition by 2030 [1]. Tackling undernutrition in India is of paramount importance, as the country accounts for two thirds of the prevalence of undernutrition globally [2].

Undernutrition is associated with poor diet among Indian children; national surveys estimate that less than 40% of the children consume an adequate diet [3, 4]. Moreover, the intake of all macronutrients and micronutrients is suboptimal [3]. For instance, only 36.6% of preschool children consumed at least 70% of the recommended daily allowance (RDA) for iron in 2016 [4].

In India, a related coexisting public health problem is anaemia, which affected 58.0% of the children in 2016 [5]. Iron deficiency is the major cause of anaemia and accounts for 76.3% of childhood anaemia nationally, albeit the estimates vary across Indian states [6]. For instance, Karnataka, a state in southern India, has a high prevalence of anaemia (51.0%); while more than half of the children (52%) have double burden of anaemia and malnutrition and are either stunted or underweight [7] .

The Indian government established a national anaemia control program based on the provision of iron and folic acid (IFA) supplements to all children [8], but a recent study concluded that the program failed to achieve the national targets due to challenges in implementation [5]. Therefore, Nguyen et al. highlighted the need for comprehensive interventions that complement IFA supplementation with counselling to improve compliance to the medication, dietary and hygienic practices [2].

**Fig. 1:**
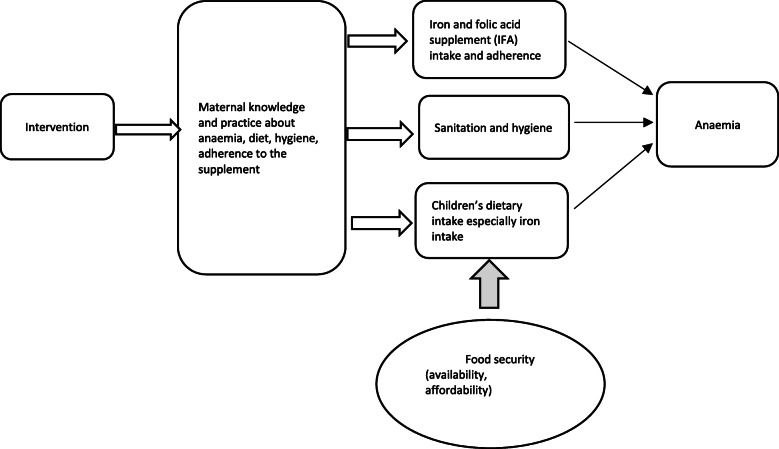
Hypothesized pathway through which the intervention interferes with the risk factors for anaemia in children. Adapted from Shet et al. [9]

In line with this suggestion, the Karnataka anaemia project was developed as a comprehensive intervention to control anaemia by addressing a chain of etiological factors that were identified using formative research [9]. The core proximal factor (iron deficiency) is seen as a function of nutritional status and hygiene, which in their turn depend heavily on maternal health literacy [9]. The proposed conceptual framework of the intervention includes improved maternal knowledge, dietary practices, and nutrients intake (especially iron) as intermediary outcomes in the pathway from the counselling to anaemia cure (Fig. 1) [9].

The intervention group received five monthly counselling sessions about anaemia, nutrition, IFA supplements and hygiene [9]. The intervention group received this counselling in addition to the usual care (20 tablets per month containing 20 mg iron and 1 mg folic acid) [9]. The comparator was anaemic children who received the standard care with IFA supplements in the same dose ^9^. The intervention was delivered proactively to mothers and caregivers of anaemic children in their homes by lay health workers called Anganwadi workers [9]. The overall effect of the Karnataka anaemia project on anaemia has been reported previously [10]. The intervention resulted in 15.1% higher anaemia cure in the intervention group compared to the control group [10]. We hypothesized that the higher anaemia cure rate among the intervention group compared to the control group was associated with an improvement in maternal knowledge, dietary practice, and children’s dietary intake. Moreover, the effect of this intervention was expected to be amplified through spill-over effect on community nutrition of all children. The expected spill-over of this lay health worker intervention is due to social proximity, as counselled and non-counselled mothers are neighbours in the same village; consequently, social interactions are expectedly high between mothers and caregivers of all the children, both anaemic and non-anaemic [11]. Therefore, this study aims to determine the participant level effects of the health worker delivered counselling on community child nutrition in Karnataka, India.

## Methods

### Study design

This is a secondary analysis of a pragmatic cluster RCT that was conducted to evaluate the effectiveness of the intervention described above [10]. The intervention protocol and primary outcomes were published previously [9, 10]. Cluster randomisation was opted for instead of individual randomisation, because the intervention was conducted by the health-care worker in charge of the Anganwadi Day-Care Centre (ADC) at the village level, thus entailing the risk of contamination in case of individual randomisation, because of the social interactions between individuals and the mode of delivery of the intervention [12]. Additionally, this design is deemed the most suitable to provide evidence on the real-world effectiveness, as it evaluates the intervention in the same setting and context for future implementation [12].

A cluster (unit of randomization) was defined as a village at Chamarajanagar district, together with the village’s ADC and the Anganwadi health worker responsible for the centre [10]. Randomisation was conducted after the clusters were stratified based on the number of children in each village (≤ 50 and > 50); since the variation in the number of children is likely to affect the quality of the counselling delivered by the Anganwadi worker.

### Study setting and time frame

This study was conducted in Chamarajanagar district of Karnataka state between November 2014 and January 2016 [10]. The ADCs are part of the national Integrated Child Development Scheme (ICDS) that was established to improve nutrition and child development [13]. Each village has an ADC run by Anganwadi worker [13]. Children aged six months to six years receive nutritional supplements, health services and informal education in the centre [13].

### Inclusion and exclusion criteria

This study included one to five-years old children living in the study area of Chamarajanagar district, Karnataka [9, 10]. Children with severe anaemia (haemoglobin less than 8 mg/dl), fever ≥38.3 °C, active infection and blood transfusion within the past three months were excluded [9, 10].

### Sample size

The sample size was calculated initially using estimated 12% effect size due to an expected 30% anaemia cure rate in the control group, and 42% anaemia cure rate in the intervention group, alpha level of 0.05, and a power of 80% [9]. The initial calculated sample was 500 children with anaemia [9]. Furthermore, design effect of 2.2 was estimated assuming Intra Cluster Correlation (ICC) of 0.05, and 25 anaemic children expected in each village, resulting in a total sample of 1100. This sample size was further inflated to account for 10% attrition; thus, the final estimate was 1220 anaemic children in total (610 anaemic children in each arm, given estimated anaemia prevalence of 50%) [9].

### Randomisation and masking

Initially, 60 out of 270 villages were selected using simple random sampling. Thereafter, the selected villages were stratified according to the number of children in each village (≤ 50 and > 50) [9, 10]. Randomisation was done simultaneously by an independent researcher who was not part of the research team. A computerised random number generator was used to randomise villages within each stratum into intervention and control group using a 1:1 ratio [9, 10]. Thereafter, the Anganwadi workers in charge of the village’s ADC were informed and trained. Data collectors and analysts in the study were blinded to the group assignment to prevent cognitive bias.

### Recruitment of clusters and participants

After randomisation, only 1284 of 1860 eligible children were screened for anaemia at baseline. This was due to the combined effects of cluster dropout (4 clusters, 2 from each group; *n* = 155) and physical absence of the participants’ families during recruitment (traveling *n* = 341; migrated/untraced *n* = 80) [10]. This led to a reduction in the study population screened at baseline (*n* = 1284) [10]. During enrolment, an entire cluster in the control group was excluded due to the lack of parental consent for blood sampling [10]. Additionally, exclusion due to severe anaemia (*n* = 48), fever (*n* = 2) and inability to participate for other reasons resulted in enrolment of 1144 participants at baseline [10].

### Participants

**Fig. 2:**
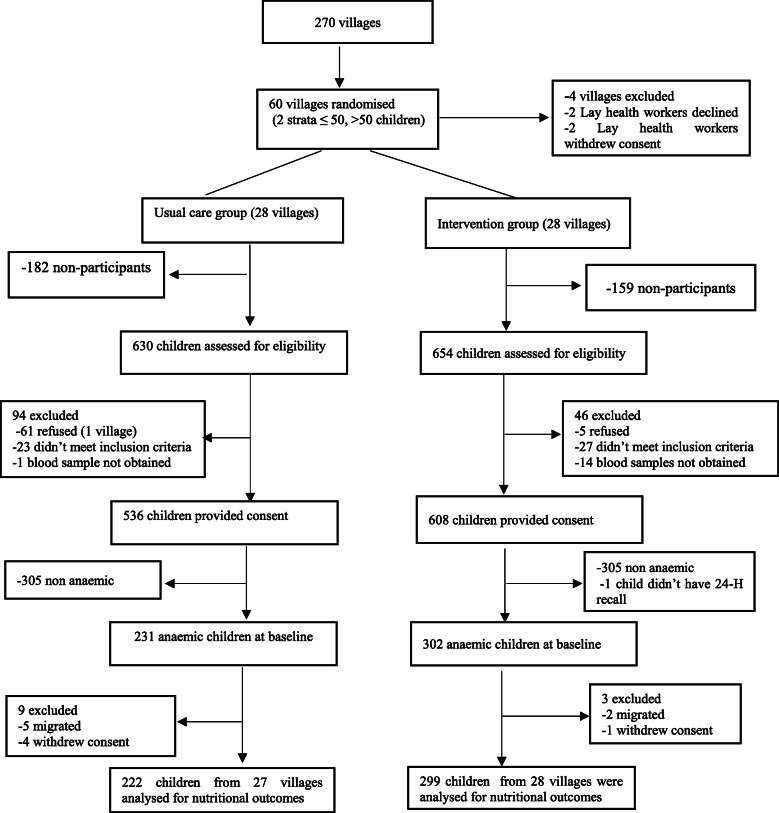
Study flowchart adapted from Shet et.al [10]

Out of the 1144 children recruited, 534 had anaemia and constituted the study population for the analysis of dietary intake at baseline [10]. The final analysis included 521 children (299 in the intervention group and 222 in the control group) retained at the 6 months follow-up. Loss to follow-up was due to migration (*n* = 1) and withdrawal of consent (*n* = 5) (Fig. 2).

On the other hand, maternal knowledge about nutrition related aspects of anaemia prevention was assessed in 1028 mothers (544 in villages randomized to the intervention and 484 in villages randomized to the control condition) after excluding 116 other caregivers. This second sample of mothers (*n* = 1028) was used to assess community-level spill over effects of the intervention.

### Intervention

The intervention consisted of counselling delivered by a health worker (Anganwadi worker) to mothers of anaemic children. The counselling included aspects of anaemia prevention including treatment and consequences of anaemia, adherence to IFA supplements, nutrition and hygiene [10]. These topics were covered in five monthly sessions over six months and the sessions were delivered in the community or at the caregivers’ home [10]. Mothers in the intervention group received the counselling in addition to IFA supplements (20 tablets per month containing 20 mg iron and 1 mg folic acid), while mothers of anaemic children in the comparison group received IFA supplements only [10].

### Outcomes

The outcomes were measured at the individual participant level. This study assessed overall child nutrition, expanding on the initial study protocol that only included nutritional aspects of anaemia prevention, i.e. dietary iron intake [9]. The decision to focus on overall child nutrition was driven by the observed double burden of anaemia and malnutrition, and the availability of data on micro and macronutrients collected from 24-H dietary recall. Therefore, this study assessed dietary intake using the change in the daily dietary intake of energy (*kcal)*, protein (g), carbohydrates (g), fat (g), vitamin C (mg), thiamine (mg), riboflavin (mg), niacin (mg), vitamin B6 (mg), folate (μg), vitamin B12 (μg), calcium (mg), and iron (mg). The intake of all nutrients was estimated at baseline, and at 6 months follow-up. Furthermore, the nutritional outcomes include the %RDA as an end nutritional goal that accounts for the difference in nutritional requirements across this age group. The %RDA is defined as “average daily dietary intake level that is sufficient to meet the nutrient requirements of nearly all, i.e. 97 to 98 percent healthy individuals in a specific life stage and gender group”. The change in %RDA from baseline to 6 months follow-up of the above-mentioned nutrients was estimated.

The educational outcome was planned in the initial study protocol and was measured as a change in maternal knowledge score about food-related aspects of anaemia prevention from baseline to 6 months follow-up. The score was derived from three questions about nutritional related aspects of anaemia prevention and ranged from a minimum of 0 to a maximum of 9.

### Data sources

Data was collected in interviews conducted at baseline and after 6 months using questionnaires and 24-H dietary recall. The questionnaires also contained information about sociodemographic factors and maternal knowledge and practice about anaemia [10]. This latter information was collected among mothers of all study participants, i.e., not only the anaemic children. A pre-coded 24-H dietary recall was used for nutritional assessment in the anaemic children; this included frequently consumed items by 1–11 years old children in the study geographic region [14]. Parents or caregivers were asked to provide information on food and beverages the child consumed in the past 24 h; predefined utensils like cups, bowls and spoons were used to make quantification standardised across respondents, and the interviewers were blinded to the group assignment [10]. Village residents prepared the most common local dishes using component ingredients that were weighed and measured by the research team [10]. Thereafter, the nutritional content of each dish was calculated based on standardised tables for nutrient value of Indian food by a nutritionist blinded to the group assignment [15]. The nutritional assessment was found to be reproducible when recalculated (10% of sample) by a hospital accredited clinical nutritionist [10].

The knowledge score questionnaire was applied to mothers during the baseline visit, and at the end of 6 months follow-up. Scores were derived from three questions related to nutritional aspects of anaemia prevention, including locally available iron-rich food (score of 6), food to be avoided with iron supplement (score of 2) and locally available food additives that enhanced iron absorption (score of 1). Score ranged from a minimum of 0 to a maximum of 9.

### Statistical analysis

The analysis was done using STATA, version 15 with the analyst blinded to the group assignment to prevent cognitive bias. Data was analysed using a modified intention- to-treat approach where all anaemic children were analysed based on the group to which they were randomised, regardless of whether or not they received the full intervention [10]. However, complete case analysis was used, i.e. children with missing data or lost to follow-up were excluded from the final analysis.

Descriptive statistics were calculated for baseline characteristics. Confidence intervals were used to make statistical inference. Mixed effects regression models were used because of the hierarchical structure of the data [16]. Linear mixed effect regression models assumed normality of distribution. The models estimated the difference in change in daily nutrients intake between the groups, and the difference in the intake as %RDA. Furthermore, the difference in change in daily nutrient intake was estimated also for the subpopulation of children of mothers’ with less than six years of education. Additionally, the linear models were used to assess the difference in change in maternal knowledge.

On the other hand, logistic mixed effect regression models were used to estimate the odds of improving and achieving the %RDA at the end of follow-up; restricting the analysis to the subgroup of anaemic children who did not achieve the %RDA at baseline. The logistic models assumed binomial distribution.

Because of the randomized design, the groups were assumed to be comparable with regards to measured predictors of the outcome [10]. Therefore, the models were not adjusted. The multi-level models were not adjusted for multiple testing, as it is proposed that they provide higher precision compared to Bonferroni-adjusted intervals [17]. The ICC was estimated after each mixed effect model.

## Results

The recruited sample was smaller than the projected one due to several reasons. Firstly, the total eligible children were less than the expected due to discrepancy in the recorded number of children in the ADC registers, and chance inclusion of villages with smaller number of children [10]. Also, more children than expected failed to be recruited, predominantly due to migration and travel. Post randomization, five clusters dropped out, but four of these were due to lay health workers nonparticipation occurring prior to study enrolment and only one cluster dropped out post enrolment due to the lack of parental permission for blood collection [10]. Exclusion of febrile and/or severely anaemic children at inception (3.65% control group vs 4.12% intervention group) further reduced the baseline sample during enrolment [10].

### Baseline characteristics of children with anaemia

The two groups resulting from random assignment were comparable with regards to sociodemographic characteristics and nutritional status at baseline with only minor differences, likely due to chance (Table 1) [10]. Anthropometric measurements revealed a high population level burden of undernutrition, but the between-group differences were small for underweight; 35.1% in the intervention group compared to 36.9% of children in the control group. Similarly, there was a high prevalence of stunting, 45.2% vs 46.8% in the intervention and control group, respectively. A smaller proportion was found to have wasting, 11.7% of children in the intervention group compared to 11.3% in the control group.

**Table 1:** Sociodemographic characteristics and nutritional status of anaemic children at baseline

Sociodemographic characteristic	Intervention (***n*** = 299) N %	Control (***n*** = 222) N %
**Child’s age in months**		
Mean (SD)	30.67 (11.9)	30.56 (12.3)
12–23	100 (33.4)	74 (33.3)
24–35	98 (32.8)	80 (36.0)
36–47	66 (22.1)	39 (17.6)
48–60	35 (11.7)	29 (13.1)
**Sex**		
Male	153 (51.2)	121 (54.5)
Female	146 (48.8)	101 (45.5)
**Maternal education in years**		
Mean (SD)	7.4 (3.8)	7.8 (4.0)
0 years	43 (14.4)	36 (16.2)
1–6	41 (13.7)	12 (5.4)
7–12	210 (70.2)	166 (74.8)
> 12	5 (1.7)	8 (3.6)
**Maternal Literacy**		
Can read and write	196 (65.6)	164 (73.9)
Cannot read and write	103 (34.4)	58 (26.1)
**DHS wealth score**		
Poorest quartile	80 (26.7)	70 (31.5)
25^th^–50th quartile	75 (25.1)	55 (24.8)
50th–75th quartile	72 (24.1)	43 (19.4)
Wealthiest quartile	72 (24.1)	54 (24.3)
**Birthweight**		
Low	41 (13.7)	36 (16.2)
Normal	258 (86.3)	186 (83.8)
**Nutritional status**		
Underweight	105 (35.1)	82 (36.9)
Stunted	135 (45.2)	104 (46.8)
Wasted	35 (11.7)	25 (11.3)

*⁕Underweight < −2 SD weight for age z score of the 2006 WHO child growth standards median.*

*⁕ Stunting < − 2 SD height for age z score of the 2006 WHO child growth standards median.*

*⁕Wasting < − 2 SD height for weight z score of the 2006 WHO child growth standards median.*

### Dietary intake of selected nutrients among children with anaemia

Daily intake of all nutrients increased in both groups from baseline to the end of follow-up, except vitamin C intake, which increased only among children in the intervention group. The confidence intervals included the value expected under the null hypothesis of no effect. Therefore, the estimates provide inconclusive evidence of a possible positive effect among children in the intervention group for all nutrients except vitamin B12 (Table 2). For nutrients related to anaemia prevention, the increase in iron intake was 0.24 mg/day (95% CI -0.67;1.15), while the increase in vitamin C intake was 4.61 mg/day (95% CI -0.69; 9.91) in the intervention group compared to the control group.

**Table 2:** Daily dietary intake of selected nutrients at the baseline, at follow-up and the between-groups difference in change between the two time points

Nutrient	Dietary intake				Difference in change between groups	
	Baseline		Follow-up			
	**Intervention (n = 299)**	**Control** **(n = 222)**	**Intervention** **(n = 299)**	**Control** **(n = 222)**	**(95% CI)**	**ICC**
Energy (kcal)	846.34	818.24	951.93	877.80	55.45 (−21.15; 136.05)	0.08
Protein (g)	22.04	21.37	26.22	24.24	1.54 (−1.30; 4.38)	0.12
Fat (g)	19.50	19.20	21.40	21.06	0.21 (−2.36; 2.78)	0.05
Carbohydrate (g)	144.86	139.61	163.08	147.36	11.91 (−1.16; 24.99)	0.07
Vit C (mg)	15.86	15.48	19.78	14.75	4.61 (−0.69; 9.91)	0.005
Thiamine (mg)	0.37	0.35	0.51	0.40	0.09 (−0.07; 0.25)	< 0.001
Riboflavin (mg)	0.46	0.47	0.56	0.53	0.03 (−0.05; 0.11)	0.06
Niacin (mg)	4.11	4.02	4.56	4.23	0.26 (−0.29; 0.82)	0.07
Vit B6 (mg)	0.46	0.45	0.53	0.48	0.04 (−0.02; 0.11)	0.07
Folate (μg)	82.56	76.40	94.78	87.95	0.64 (−11.29; 12.58)	0.08
Vit B12 (μg)	0.56	0.59	0.65	0.72	−0.04 (−0.20; 0.12)	0.05
Calcium (mg)	293.83	289.27	339.50	327.84	8.66 (−35.07; 52.38)	0.08
Iron (mg)	4.58	4.22	5.39	4.78	0.24 (−0.67; 1.15)	0.04

*CI = confidence interval, g = gram, ICC = intra cluster correlation, kcal = kilocalorie, mg = milligram, μg = microgram, Vit = vitamin*

*The difference in change is calculated using linear mixed effect regression models by subtracting change in the control group from change in the intervention group. A positive difference means the intake increased to a higher extent among the intervention group compared to the control group from baseline to end of follow-up*

The average daily allowance of all nutrients expressed as %RDA increased in both groups from baseline to the end of follow-up except for vitamin C intake, which decreased among children in the control group. The intake of most minerals and vitamins for both groups remained below the %RDA (Table 3). For instance, the daily iron intake increased from 45.0% at baseline to 50.5% of the %RDA at the end of follow-up, and the daily intake of vitamin C increased from 39.6 to 49.4%. On the contrary, the dietary intake of protein and folate were at or above the %RDA at both time points (Table 3).

**Table 3:** Average intake of selected nutrients expressed as % RDA at baseline, at follow-up and between-groups difference in change between the two time points

Nutrient	Mean intake in % RDA Baseline Follow-up				Difference in change between groups	
	Intervention (n = 299)	Control (n = 222)	Intervention (n = 299)	Control (n = 222)	(95% CI)	ICC
Energy	77.38	74.72	85.51	78.72	4.85 (− 2.45; 12.16)	0.070
Protein	123.97	120.80	143.67	133.45	8.01 (−7.65; 23.68)	0.103
Vit C	39.65	38.69	49.46	36.88	11.52(−1.73; 24.77)	0.005
Thiamine	71.21	67.46	95.67	74.24	17.33(−14.98; 49.63)	< 0.001
Riboflavin	74.97	74.99	88.26	83.92	4.32(−8.53; 17.16)	0.054
Niacin	49.07	48.23	53.23	49.58	3.15(−3.18; 9.48)	0.062
Vit B6	51.41	50.06	59.35	53.62	4.75(−2.54; 12.03)	0.072
Folate	95.50	89.12	106.70	99.10	0.93(−12.42; 14.29)	0.092
Vit B12	55.65	58.56	64.85	71.94	−4.17(−20.11; 11.77)	0.062
Calcium	48.97	48.21	56.58	54.64	1.44(−5.85; 8.73)	0.083
Iron	38.31	44.79	55.25	49.27	2.24(−7.43; 11.91)	0.043

*The difference in change is calculated using linear mixed effect regression model by subtracting change in the control group from change in the intervention group. A positive difference means the intake among the intervention group increased to a higher extent than the control group between baseline and end of follow-up*

Among the children who did not achieve the %RDA at baseline, the odds of achieving the %RDA for iron at the end of follow-up was 2.13 (95%CI 0.74; 6.13) for the intervention group compared to the control group. Furthermore, the odds of achieving the %RDA for vitamin C at the end of follow-up was 1.24 (95%CI 0.48; 3.24) for the intervention group compared to the control group (Supplementary Table 1).

The estimates among children whose mothers had less than 6 years of education were not substantially different from those in the whole group, with confidence intervals that included the value expected under the null hypothesis. The difference in change in energy intake from baseline to 6 months follow-up was 46.82 kcal (95%CI -92.28; 185.91), protein intake was 2.38 g (95%CI -2.50; 7.26) higher in the intervention group compared to the control group. The estimates for nutrients related to anaemia prevention also provide inconclusive evidence of a possible effect. For instance, the difference in change in iron intake from baseline to 6 months follow-up was 0.68 mg/day (95%CI -0.36;1.71). Furthermore, the difference in change in vitamin C intake was 4.88 mg/day (95% CI -10.24; 20.01) higher in the intervention group compared to the control group (Supplementary Table 2).

### Maternal knowledge

The difference in change in the knowledge score was 0.15 (95% CI -0.05; 0.36) points in the intervention group compared to the control group.

## Discussion

The aim of this study was to determine whether maternal education and counselling by a community health worker attained measurable effects on childhood nutritional outcomes. The findings of this study provide inconclusive evidence of the intervention effect on dietary intake among children. This can be attributed to the small sample size, as this is a secondary analysis of a cluster RCT that was not initially powered to detect differences in dietary intake. Moreover, this was a complex intervention where counselling primarily focused on anaemia prevention rather than on overall nutrition. In fact, the nutrition counselling in this intervention covered dietary diversification as a mean of nutritional anaemia prevention rather than addressing global childhood nutritional practices, in order to target malnutrition. Furthermore, in this rural community, economical constraints might have had a much greater influence on dietary quality than the health literacy acquired through the intervention [18].

There is also inconclusive evidence of the effect of the intervention on the intake of nutrients related to anaemia that were the primary focus of the counselling, namely, the change in iron and vitamin C intake. These findings indicate that targeted nutritional counselling might not be effective, raising the question of the effectiveness of less specific interventions. Besides, the counselling encouraged the inclusion of locally available sources of vitamin C, e.g. lemon, which despite having a relatively low vitamin C content compared to other seasonal fruit, e.g. guava (48 mg per 100 g edible portion compared to 222 mg per 100 g edible portion) is both economically affordable and available all year round [19]. Nevertheless, the cost of iron rich food with highly bioavailable iron, e.g. animal protein might have been a barrier to additional dietary diversification leading to reliance on supplemental IFA.

An increase in dietary intake is noticeable in all nutrients except vitamin c, from baseline to the end of follow-up among all anaemic children in the study sample. Obviously, it could be partly attributed to the maturation effect (growth) which increases children’s dietary requirement and consequently dietary intake. However, this explanation does not reconcile with the improvement in %RDA that takes age into consideration. Additionally, enrolment into the study could have enhanced caregivers’ awareness of nutritional aspects and consequently resulted in the change in nutritional practices regardless of the group assignment, or else to faulty and inflated dietary reports. A systematic review concluded that this phenomenon leads to overestimated effects of behavioural interventions, especially those interventions involving measures of self-reported dietary intake [20]. However, in this trial both study groups received similar attention apart from the intervention, thus mitigating the effects of this bias.

In this study we also examined the average dietary intake in %RDA as the end inspirational goal of nutritional counselling. Although the average intake in %RDA of all nutrients except vitamin C suggests a possible positive trend in both groups from baseline to the end of follow-up, the intake of many micronutrients remained below the %RDA. Studies have highlighted that economical constraints might explain micronutrients deficiency, as it was reported that household food security is only achieved for macronutrients in India [2, 18, 21]. The intake of protein in this sample (above %RDA) further supports this explanation. However, the estimates appear to suggest a positive direction of effect on the subgroup of children who did not achieve the %RDA at baseline.

When comparing the findings of this study to published literature on nutritional counselling, similarities and differences between the interventions must be considered carefully [17, 21–24]. Of note is that most of the studies reported on primary outcomes of interventions powered to detect a difference in dietary intake [18, 22–24]. In contrast, this study investigated secondary outcomes of an intervention that was powered to detect difference in anaemia cure rate (primary outcome).

For instance, Vazir et al. conducted a cluster RCT in rural India and reported that intake of micronutrients remained below the %RDA, while the intake of protein was above the %RDA similar to our findings [18]. The discrepancy in the effect between our study and Vazir et al. might be due to either differences in follow-up duration (12 months) or study outcome, as the intervention focused on improving overall nutrition [18].

On the other hand, differences in intervention components might explain the discrepancy between our findings and findings of studies conducted in different settings [21–23]. For instance, a Malawian study by Gelli et al. included an agricultural component, cooking demonstrations in addition to nutritional counselling [22]. The findings of Gelli et al. along with those from this trial lend support to the notion that availability of economically affordable food is crucial to attaining robust improvements in child nutritional outcomes. Therefore, in settings where food insecurity and economical constraints affect the quality of diet, nutrition interventions that include measures such as microfinancing, food supplements or vouchers are more likely to yield significant improvements in children’s nutritional outcomes. This notion is further supported by the findings of a microfinancing intervention in India that reported improvement in nutrition among children under the age of five [25].

Although inconclusive, the estimation of the effect of the intervention on children whose mothers received less than six years of education deserves attention in the light of the potential of reducing the socially determined gap in health. It also implies that Anganwadi workers delivered comprehensible messages even among less educated mothers. It might also reflect the nature of the intervention that sought to achieve behaviour change through improved maternal self-efficacy and did not focus solely on improving maternal knowledge.

Along with this observation, we also noted that this intervention did not spill over to the whole community concerning maternal knowledge about nutritional aspects of anaemia prevention. However, this lack of knowledge change might also be due to the insensitivity of the scale used in the study, that included only three questions about food related aspects of anaemia prevention with a maximum possible score of nine. Another likely explanation is that Anganwadi workers might be more trained to enhance skills rather than knowledge. Moreover, the Anganwadi workers are overburdened, focusing more on distribution of food and initiation of breastfeeding, and less on child-focused nutrition counselling [26].

The published literature on the effect of nutritional counselling on maternal knowledge reports varying results [18, 22, 23, 27, 28]. A significant improvement in maternal knowledge was reported in studies in Ethiopia and Kenya, contrary to our findings, which might be attributed to the duration and intensity of these interventions and their sole focus on nutrition [24, 27, 28]. Other studies are conflicting and found that nutrition counselling had no effect on maternal knowledge [18, 22]. For instance, Vazir et al. concluded that nutrition counselling had little effect on improving complementary feeding knowledge [18]. However, these investigators also recognized that the scale they used might have been insensitive to small effect sizes; a similar concern as in this study [18].

Published literature on spill-over effects of interventions due to social proximity is scant [11, 29]. On one hand, a systematic review concluded that the evidence supporting spill-over benefits of study outcomes through social proximity are scarce, probably due to the complexity of behavioural interventions and the socio-cultural factors involved in the settings in which they are tested [11]. On the other hand, a behavior change communication in Bangladesh reported a positive spill-over effect on the knowledge of neighbouring non-participants [28]. However, a more sensitive knowledge scale with 14 points was employed in the study, possibly explaining the difference from our findings [29]. Other factors that might influence the uptake of behavioral interventions are the inclusion of cash, food supplies, and other benefits in addition to behavior change communication [29].

### Strength and limitations

The main strength of this study is the experimental pragmatic design leading to comparable groups and results that are not likely to be biased by confounding, while preserving the “real life” characteristics of the setting [9]. Moreover, the Anganwadi health workers were not trained in the same centre where they worked to minimize contamination, and data collectors and analysts were blinded to minimize cognitive bias [10]. In line with the pragmatic nature of the intervention, the findings of this study might be generalisable to rural India where the ADC centres are present and the Anganwadi workers have similar tasks and responsibilities. However, the content of the counselling probably needs to be adapted to the local context of each area since the intervention promotes the consumption of locally available food.

One limitation of the 24-H dietary recall is that it possibly overestimates energy and micronutrients intake in toddlers and young children [30]. However, study participants were asked to recall the last typical day to maximize the chance that the reported intake accurately reflected the child’s habitual diet [31]. Moreover, as villages weren’t blinded to the group assignment, differential misclassification cannot be ruled out. However, given similarities of intervention implementation between the experimental groups (the only difference being that mothers of anaemic children in the interventional villages received counselling), non-differential responses were unlikely.

Another limitation is the use of a rather crude scale to assess maternal knowledge about food related aspects of anaemia prevention. This scale was not validated and might have lacked sensitivity to capture modest changes in maternal knowledge. However, a trade-off was made between the comprehensiveness and usability of the scale.

This study benefited from a low attrition rate (2.2%). However, in this study four clusters dropped post randomisation, although this was balanced between the two groups, as two clusters were lost from each group due to lay health worker refusing to participate. Moreover, mothers in one village refused to participate due to lack of fathers’ consent [10]. Finally, this study was inadequately powered to detect differences in nutrient intake that might have occurred as a result of exposure to the intervention. Importantly, this analysis disentangles the effect of different components of a complex intervention and highlights the potential additive value of nutritional counselling.

## Conclusion

The effect of parental counselling on child nutrition parameters are inconclusive, possibly due to limitations imposed by the sample size. These results highlight the need to develop multi-component anaemia-prevention interventions that are adequately powered to detect secondary outcomes and may allow the study of the distinct effects of different components. Moreover, in settings where food insecurity and economical constraints affect the quality of diet; nutrition interventions need to include food supplements or vouchers to facilitate the availability of nutritious food.

## Supplementary Information

**Additional file 1.**

## Data Availability

Deidentified participant data used and/or analysed during the current study are available from the corresponding author israa.ali@ki.se on reasonable request.

## Abbreviations

ADC: Anganwadi Day-care Centre
CI: Confidence Interval
g: Gram
ICC: Intra Cluster Correlation
ICDS: Integrated Child Development Services
IFA: Iron and Folic Acid
kcal: Kilocalorie
mg: Milligram
μg: Microgram
RCT: Randomised Controlled Trial
RDA: Recommended Daily Allowance
SDGs: Sustainable Development Goals
WHO: World Health Organisation
