# Supplementary material for: Effect of a maternal counselling intervention delivered by community health workers on child nutrition: secondary analysis of a cluster randomised controlled trial in India
Source: BMC Public Health. 2021 Nov 5;21:2015. doi: 10.1186/s12889-021-11998-w (PMC8571833; doi:10.1186/s12889-021-11998-w)
Supplement: Supplementary file 1 — Additional file 1. [file 12889_2021_11998_MOESM1_ESM.docx]

**Supplementary Table 1: Odds ratios (OR) and 95% confidence interval of attaining the RDA at follow-up among participants not meeting the RDA at baseline, by experimental group.**

| **Nutrient** | **Children not achieving RDA** | | | | | **Odds ratio (95% CI) of attaining RDA** | |
| --- | --- | --- | --- | --- | --- | --- | --- |
|  | **Baseline** | | **Follow up** | | |  |  |
|  | **Intervention**  **N %** | **Control**  **N %** | | **Intervention**  **N %** | **Control**  **N %** | **Intervention** | **Control** |
| Energy | 226 (74.8) | 191 (82.7) | | 224 (74.7) | 184 (82.9) | 1.67 (0.97; 2.86) | 1.00  (ref) |
| Protein | 90 (29.8) | 71 (30.7) | | 52 (17.3) | 49 (22.1) | 1.12 (0.49; 2.56) | 1.00 |
| Vit C | 287 (95.0) | 216 (93.5) | | 284 (94.7) | 213 (95.9) | 1.24 (0.48; 3.24) | 1.00 |
| Thiamine | 254 (84.1) | 198 (85.7) | | 239 (79.7) | 188 (84.7) | 1.30(0.62; 2.71) | 1.00 |
| Riboflavin | 218 (72.2) | 164 (71.0) | | 190 (63.3) | 149 (67.1) | 1.20(0.65; 2.21) | 1.00 |
| Niacin | 291 (96.4) | 219 (94.8) | | 293 (97.7) | 218 (98.2) | 1.40(0.30; 6.47) | 1.00 |
| Vit B6 | 286 (94.7) | 219 (94.8) | | 269 (89.7) | 214 (96.4) | 3.20(0.74; 13.88) | 1.00 |
| Folate | 162 (53.6) | 134 (58.0) | | 147 (49.0) | 114 (51.4) | 0.87(0.53; 1.42) | 1.00 |
| Vit B12 | 255 (84.4) | 201 (87.0) | | 253 (84.3) | 185 (83.3) | 0.80 (0.32;1.98) | 1.00 |
| Calcium | 290 (96.0) | 221 (95.7) | | 278 (92.7) | 213 (95.9) | 1.91(0.65; 5.60) | 1.00 |
| Iron | 289 (95.7) | 223 (96.5) | | 286 (95.3) | 216 (97.3) | 2.13(0.74; 6.13) | 1.00 |

** CI= confidence interval, RDA= recommended daily allowance, Vit= vitamin.*

**Supplementary Table 2: Daily dietary intake of selected nutrients among anaemic children whose mothers had less than 6 years of education at baseline, end of follow up and the between-groups difference in the mean change between the two time points**

| **Nutrient** | **Dietary Intake** | | | | **Difference in change between groups** |  |
| --- | --- | --- | --- | --- | --- | --- |
|  | **Baseline** | | **Follow up** | |  |  |
|  | **Intervention** | **Control** | **Intervention** | **Control** | **(95% CI)** | **ICC** |
| Energy (kcal) | 873.39 | 812.44 | 973.57 | 873.64 | 46.82 (-92.28; 185.91) | 0.17 |
| Protein (g) | 23.15 | 22.10 | 27.26 | 24.13 | 2.38 (-2.50; 7.26) | 0.22 |
| Fat (g) | 19.75 | 17.36 | 21.58 | 20.44 | -1.15 (-5.31; 3.01) | 0.07 |
| Carbohydrate (g) | 150.67 | 141.68 | 167.48 | 147.53 | 12.06(-11.02;35.13) | 0.18 |
| Vit C (mg) | 13.88 | 11.04 | 21.32 | 13.49 | 4.88 (-10.24; 20.01) | <0.001 |
| Thiamine (mg) | 0.36 | 0.36 | 0.44 | 0.39 | 0.05 (-0.04; 0.13) | 0.21 |
| Riboflavin (mg) | 0.46 | 0.45 | 0.53 | 0.54 | -0.18 (-0.12; 0.09) | 0.01 |
| Niacin (mg) | 4.02 | 3.91 | 4.72 | 4.03 | 0.66 (-0.11; 1.44) | 0.18 |
| Vit B6 (mg) | 0.46 | 0.46 | 0.54 | 0.48 | 0.08 (-0.04; 0.20) | 0.27 |
| Folate (µg) | 88.20 | 75.41 | 98.80 | 90.66 | -3.33 (-23.21; 16.55) | 0.11 |
| Vit B12 (µg) | 0.55 | 0.53 | 0.62 | 0.70 | -0.08 (-0.26; 0.09) | 0.08 |
| Calcium (mg) | 300.41 | 275.93 | 324.11 | 338.25 | -34.52(-100.76; 31.72) | 0.09 |
| Iron (mg) | 4.43 | 4.24 | 5.18 | 4.35 | 0.68 (-0.36; 1.71) | 0.19 |

** CI= confidence interval, g=gram, ICC= intra cluster correlation, kcal= kilocalorie, mg= milligram, µg= microgram, Vit= vitamin*

***** *The difference in change is calculated using mixed linear regression models by subtracting change in the control group from change in the intervention group. A positive difference means the intervention group improved to a higher extent from baseline to the end of follow.*
